# Antigenotoxic Studies of Different Substances to Reduce the DNA Damage Induced by Aflatoxin B_1_ and Ochratoxin A

**DOI:** 10.3390/toxins2040738

**Published:** 2010-04-19

**Authors:** Eduardo Madrigal-Santillán, José A. Morales-González, Nancy Vargas-Mendoza, Patricia Reyes-Ramírez, Sandra Cruz-Jaime, Teresa Sumaya-Martínez, Ricardo Pérez-Pastén, Eduardo Madrigal-Bujaidar

**Affiliations:** 1 Instituto de Ciencias de la Salud, Universidad Autónoma del Estado de Hidalgo., Ex-Hacienda de la Concepción. Tilcuautla. Pachuca de Soto, Hidalgo. CP 42080, México; Email: jmorales101@yahoo.com.mx (J.A.M.); nvargas_mendoza@hotmail.com (N.V.); pattyreyes_1@hotmail.com (P.R.); crzjms@msn.com (S.C.); teresumaya@hotmail.com (T.S.); 2 Laboratorio de Genética, Escuela Nacional de Ciencias Biológicas, I.P.N., Av. Wilfrido Massieu. Unidad A. López Mateos. Zacatenco. Col Lindavista. D.F. CP 07738, México; Email: eduardo.madrigal@lycos.com (E.M.B.); 3 Laboratorio de Toxicología Preclínica, Escuela Nacional de Ciencias Biológicas, I.P.N., Av. Wilfrido Massieu. Unidad A. López Mateos. Zacatenco. Col Lindavista. D.F. CP 07738, México; Email: pastenrich@yahoo.com.mx (R.P.)

**Keywords:** aflatoxin B_1_, ochratoxin A, antigenotoxic, DNA damage

## Abstract

Mycotoxins are produced mainly by the mycelial structure of filamentous fungi, or more specifically, molds. These secondary metabolites are synthesized during the end of the exponential growth phase and appear to have no biochemical significance in fungal growth and development. The contamination of foods and feeds with mycotoxins is a significant problem for the adverse effects on humans, animals, and crops that result in illnesses and economic losses. The toxic effect of the ingestion of mycotoxins in humans and animals depends on a number of factors including intake levels, duration of exposure, toxin species, mechanisms of action, metabolism, and defense mechanisms. In general, the consumption of contaminated food and feed with mycotoxin induces to neurotoxic, immunosuppressive, teratogenic, mutagenic, and carcinogenic effect in humans and/or animals. The most significant mycotoxins in terms of public health and agronomic perspective include the aflatoxins, ochratoxin A (OTA), trichothecenes, fumonisins, patulin, and the ergot alkaloids. Due to the detrimental effects of these mycotoxins, several strategies have been developed in order to reduce the risk of exposure. These include the degradation, destruction, inactivation or removal of mycotoxins through chemical, physical and biological methods. However, the results obtained with these methods have not been optimal, because they may change the organoleptic characteristics and nutritional values of food. Another alternative strategy to prevent or reduce the toxic effects of mycotoxins is by applying antimutagenic agents. These substances act according to several extra- or intracellular mechanisms, their main goal being to avoid the interaction of mycotoxins with DNA; as a consequence of their action, these agents would inhibit mutagenesis and carcinogenesis. This article reviews the main strategies used to control AFB_1_ and ochratoxin A and contains an analysis of some antigenotoxic substances that reduce the DNA damage caused by these mycotoxins.

## 1. Introduction

Mycotoxins are structurally diverse groups composed mainly of small molecular weight compounds. These compounds are produced mainly by the mycelial structure of filamentous fungi, or more specifically, the molds. Mycotoxins are secondary metabolites synthesized during the end of the exponential phase of growth and appear to have no biological significance with respect to mould growth/development or competitiveness, but when ingested by higher vertebrates and other animals cause diseases called mycotoxicoses [1,2]. The metabolites are found in a wide range of countries, feeds and foods [3]. They are toxic to mammals, poultry, and fish [2,4,5]. The toxic effect of mycotoxin ingestion in both humans and animals depends on a number of factors including intake levels, the toxicity of the compound, duration of exposure (acute or chronic), the body weight of the individual, the presence of other mycotoxins (synergistic effects), mechanisms of action, metabolism, and defense mechanisms [1,6,7]. Metabolism and defense mechanisms are important factors in understanding mycotoxin toxicity in specific species or individual animals. Specificity of such mechanisms is well demonstrated in the significant difference between ruminants and non ruminants in handling mycotoxins. Ruminants have generally been more resistant to the adverse effects of mycotoxins. This is because the rumen microbiota is capable of degrading mycotoxins [8]. Starting with the discovery of the aflatoxins in the early 1960s, the isolation of mycotoxins from food has led to the identification of over 100 toxigenic fungi and more than 400 mycotoxins [1,9]. These toxins account for millions of dollars annually in losses worldwide in human and animals health, and condemned agricultural products. Different factors contributing to the presence or production of mycotoxins in foods or feeds include storage, environmental, and ecological conditions. Often times most factors are beyond human control [8].

Nowadays, mycotoxins with carcinogenic potential in experimental animal models include aflatoxins, ochratoxin, sterigmatocystin, fumonisin, zearalenone, citrinin, patulin, and luteoskyrin [9]. These carcinogenic mycotoxins are DNA damaging agents, with the only exception of fumonisins [10]. They may produce cancer by interference with signal transduction pathways [11].

Many cereals, oil seeds, tree nuts, and dehydrated fruits are susceptible to fungus contamination and mycotoxin production. Under laboratory conditions at least 300 mycotoxins produced by fungal culture broth have been chemically characterized. Fortunately, only about 20 mycotoxins are known to be present in food at significant levels. These toxins are mainly produced by five genera of fungi: *Aspergillus, Penicillium, Fusarium, Alternaria*, and *Claviceps* [12].

This review attempts to briefly summarize the current available data of the main biological properties and mechanisms of DNA damage caused by aflatoxin B_1_ and ochratoxin A. It also reviews the main strategies used to control of these mycotoxins.

## 2. Generalities of Aflatoxins

**Figure 1:**
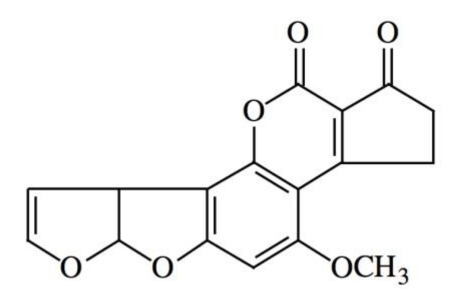
Chemical structure of aflatoxin B_1 _(AFB_1_).

Aflatoxins were first isolated some 40 years ago after outbreaks of disease and death in turkeys and of cancer in rainbow trout fed on rations formulated from peanut and cottonseed meals. These secondary metabolites are a group of closely related difuranocoumarin compounds produced by several strains of filamentous fungi, mainly by *Aspergillus flavus* and *Aspergillus parasiticus* [12,13]. Aflatoxins may produce considerable economic losses by attacking different stages of sowing and industrialization of different agricultural and dairy products. They can contaminate a great number of crops used for human and animal consumption, for example, corn, peanut, sorghum, rice, wheat, and nut as well as various milk-made products [14]. However, the range of contaminated products differs depending on the country: for example, in Japan, aflatoxins were detected in about 50% of peanut butter and bitter chocolate samples, while their presence was not found in corn products; in contrast, a study in China reported contamination of 70% of corn products [15,16]. In Mexico and other countries, corn is a cereal used as the main component of several meals. Studies have shown that in the cultivation or storage processes of this grain there exist different levels of aflatoxin contamination, particularly with aflatoxin B_1 _(AFB_1_) (Figure 1) [14,17]. This chemical is the most potent natural mutagen and carcinogen known and is usually the major aflatoxin produced by toxigenic strains. It is also the best studied: in a large percentage of the papers published, the term aflatoxin can be construed to mean aflatoxin B_1_ [2].

## 3. DNA Damage by Aflatoxin B_1_ (AFB_1_)

In regards to its genotoxic effects, AFB_1_ has been evaluated through *in vitro* and *in vivo* systems which have shown an increase in the rate of DNA adducts, histidine revertants, chromosomal aberrations, micronucleus and sister chromatid exchanges [18,19,20,21,22]. AFB_1_ is a powerful carcinogen for humans and many animal species, including rodents, non-human primates, and fish [5,23]. The main target of this carcinogen is the liver, although tumors may also develop in other organs, such as the lungs, kidney and colon [9].

There are substantial differences in species susceptibility. Moreover, within a given species, the magnitude of the response is influenced by age, sex, weight, diet, exposure to infectious agents, and the presence of other mycotoxins and pharmacologically active substances. Diverse studies on aflatoxin toxicity have been conducted, mostly concerning laboratory models or agriculturally important species [2,24]. Cytochrome P450 enzymes convert aflatoxin B_1_ to the reactive 8,9-epoxide form (AFBO), which is capable of binding to both DNA and proteins [24]. Mechanistically, the AFBO metabolite binds covalently to guanine to form the N^7^-guanine-AFBO adduct, an event which could cause the diverse expressions that characterize the AFB_1_ genotoxicity [24,25]. Moreover, aflatoxin B_1_-DNA adducts can result in GC to TA transversions. A reactive glutathione S-transferase system found in the cytosol and microsomes catalyzes the conjugation of activated aflatoxins with reduced glutathione, leading to the excretion of aflatoxin [26]. Variation in the level of the glutathione transferase system as well as variations in the cytochrome P450 system are thought to contribute to the differences observed in interspecies aflatoxin susceptibility [24,27].

### 3.1. Antimutagenic Strategies Used to Control the AFB_1_ Damage

There is some evidence suggesting that there may be a potential risk that AFB_1_ can cause cancer. Therefore since 1993, The International Agency for Research on Cancer (IARC) has classified it as a high potential carcinogenic agent (Class I) [28]. The potential risk has promoted the evaluation of norms that regulate the quantity of AFB_1_ in foods. 

One of the first authors that evaluated these norms was Labuza, who in 1983, examined three aspects of mycotoxins in food in the USA: (a) relevant laws, (b) the Food and Drug Administration (FDA) guidelines with respect to the law and (c) the courts interpretation of these laws. Also presented several cases of regulations applied to interstate shipments of corn. FDA analyzes raw agricultural products for aflatoxin through the compliance program which objectives are: the collection and analysis of food and feeds to determine regulatory levels; to remove from interstate commerce food that contains violative aflatoxin levels and to determine potential problems and control measures. Nevertheless, no information on the regulatory issues dealing with mycotoxins in international trade of corn was presented. Furthermore, the USA was one the first countries to introduce legislation regulating on aflatoxin levels in food and feed [29]. Labuza’s publication leads to the adoption, expansion, and changes in regulations concerning mycotoxins in many countries. An international inquiry on mycotoxins was initiated by the National Institute for Public Health and the Environment. Dutch embassies around the world were requested to gather up-to-date information about mycotoxin regulations from local authorities. At least 99 countries had regulations concerning mycotoxins in food and feed in 2003. All of them have at least regulatory limits for aflatoxin B_1_ contents and specific regulations exist for other mycotoxins as well. The maximum tolerated levels for aflatoxin B_1_ in food are from 1 to 20 µg/kg. The most frequently occurring limit is 4 µg/kg, and this limit is applied in the 29 countries which follow the harmonized regulations of the European Free Trade Association (EFTA) and the European Union (EU). The 20 µg/kg limit is applied in 17 countries: half of them are in Latin America, United States and Africa [30]. Although security standards have been implemented, the damage caused by AFB_1_ is still important and serious. In our country, the contamination caused by this toxin can affect food and sweets made of corn, cereals and oilseeds. As well as meat of chicken, pork and other animal-derived products (milk, eggs or cheese) [31,32,33].

Several strategies have been developed in order to reduce the risk of aflatoxin exposure. These include the degradation, destruction, inactivation or removal of mycotoxins through chemical and physical methods (Table 1). However, the results obtained with these methods have not been optimal, because they change the organoleptic characteristics and nutritional values of food [34,35,36]. Another alternative strategy to prevent or reduce the genotoxic effects of AFB_1_ is by applying antimutagenic agents. These substances act according to different mechanisms being their main goal to avoid the interaction of AFB_1_ with DNA. In general, the antigenotoxic agents may act blocking the mutation and cancer initiation in the extracellular environment or in nontarget cells (inhibition of uptake of mutagens/carcinogens, inhibition of the endogenous formation of carcinogens, or favoring the adsorption of protective agents) as well as by intracellular mechanisms such as the inhibition of mutation and cancer initiation in target cells or the tumor promotion. As a consequence of their action, these agents would inhibit mutagenesis and carcinogenesis [37].

**Table 1:** Possible strategies to avoid mycotoxin contamination of agricultural products.

Methods	Technique	Example	Reference
Physical	Inactivation by heat	Vapor pressure	[38]
		Microwave treatment	[39]
		Nixtamalization	[40]
	Inactivation by radiation	Ultraviolet light	[39]
		Radiation gamma	[1,41]
	Elimination by adsorbent substances	Zeolites	[1]
		Bentonites	[39]
		Aluminosilicates	[42]
Chemical	Extraction by solvents organics	Ethanol 95%	[43]
		Acetone 90%	[39]
	Chemical destruction	Hexane-ethanol	[43]
		Hydrogen peroxide	[44]
		Ammonium hydroxyde	[45,46]
		Methylamine	[39]
		Sodium hypochlorite	[44]

### 3.2. *In Vitro* Studies

In 1994 Singh *et al*. studied the effect of piperine on the cytotoxicity of aflatoxin B1 (AFB_1_) in rat hepatoma cells H4IIEC3/G-(H4IIE) and demonstrated that AFB_1 _inhibited the growth of H4IIE cells. Likewise, piperine reduced the AFB_1_-induced formation of micronuclei in a concentration-dependent manner [47]. In another study about the protective effect of food additives by Soni *et al.* (1997) it was demonstrated that all the additives studied had an important inhibition against mutation, the most effective was turmeric and curcumin that inhibited mutation frequency by more than 80% at concentrations of 2 µg/plate in Salmonella tester strains TA 98 and TA 100 [48]. In 1996 Loarca-Piña used the Salmonella microsuspension assay to examine the antimutagenicity of ellagic acid (EA) against this potent mycotoxin using tester strains TA98 and TA100, the results of the sequential incubation studies support the hypothesis that one mechanism of inhibition could involve the formation of a chemical complex between EA and AFB_1_ [49]. On the other hand, in 1992 Decoudu *et al.* studied the effect of vitamin A dietary intake on in vitro and showed the relationship between vitamin A and AFB_1_ [42]. It demonstrated the activities of metabolizing enzymes which specifically activate or deactivate AFB_1_ and has decreased significantly in vitamin A-deficient animals [47,48,49,50,51].

### 3.3. *In Vivo* Studies

Considering the above results, the next stage has been to confirm the effectiveness of antimutagenic agents in eukaryotic organisms, particularly in mammals. Nowadays, these studies have been used as experimental models on mice and rats (Table 2). Those studies began in the nineties, and they evaluated the antimutagenic activity of some vitamins (thiamine, riboflavin, niacin and folic acid), extract of coffee and some carotenoids. Cytogenetic tests have been used (micronucleus assay) and evaluation of adducts and DNA breaks [50,52,53,54]. In general, the results have been favorable, showing different inhibitions of genotoxicity, which depend on the dose, route of administration and the exposure time.

Since 1993, our laboratory has evaluated the potential of ammonium hydroxide used to reduce the frequency of micronuclei (MN) and sister chromatid exchange (SCEs) in mice fed with AFB_1_ contaminated corn. The micronucleus intracytoplasmic of chromatin corpuscles formed from the breakup of acentric chromosome fragments or chromosomes with an anaphase lag. SCEs are chromosomal damage referred to the production of homologous segments exchanges between sister chromatids in any chromosome and both are useful parameters to detect DNA damage.

The experiment lasted eight weeks. During four weeks the animals were fed with a balanced diet and corn. One animal of this group was intoxicated by AFB_1_ contaminated diet (15 ppb). AFB_1_ and ammonia was added to other group and only ammonia was added to the last. In the last four weeks of the experiment, the animals were only fed with a balanced diet and uncontaminated maize [55]. The MN were quantified every week with a erythrocyte normochromic of peripheral blood and the results showed a significant reduction in MN since the first week of treatment, reaching a maximum treatment effect of the 60% in the fourth week. The SCEs were obtained from bone marrow and quantified in the fourth and eighth weeks. An inhibition of 55% was observed in the fourth week. At the end of the following four weeks the groups treated with AFB_1_ and AFB_1_ plus ammonia showed incomplete recovery of genotoxic damage. In this experiment the antigenotoxic effect of ammonium was attributed to the production of the corresponding hydroxy acid.

**Table 2:** Summary of various antigenotoxic agents used in mice or rats treated with AFB_1_.

Year	Biological model	Antimutagen	Type of study	Observation	Inhibition (%)	Reference
1991	Mice	Coffe	Acute	Micronucleus	60	[53]
1992	Rat	Vitamin A	Subchronic	Single cell electrophoresis	50	[50]
1992	Mice	Vitamins: thiamine, riboflavin, niacin, and folic acid	Subchronic	Micronucleus	60	[54]
1993	Mice	Ammonium hydroxyde	Subchronic	Micronucleus and Sister chromatid exchanges (SCEs)	60	[55]
1998	Rat	Carotenoids		Quantification of adducts	65	[52]
1998	Mice	*S. cerevisiae*	Subchronic	Micronucleus	70	[22]
2007	Mice	Mannan	Subchronic	Micronucleus	50	[56]
2009	Mice	Mannan	Acute	single cell electrophoresis	60	[25]

### 3.4. Antimutagenic Effect of Yeast

Probiotics are organisms and substances that contribute to intestinal microbial balance. They have the capacity to capture microorganisms pathogens. Among them are the lactobacilli and yeasts [57]. Based on the usefulness of the model used with ammonium and considering that probiotics have shown adsorbent, immunostimulants and antigenotóxic effects, our laboratory continued studies using *Saccharomyces cerevisiae* (*Sc*) as antimutagen.

**Figure 2:**
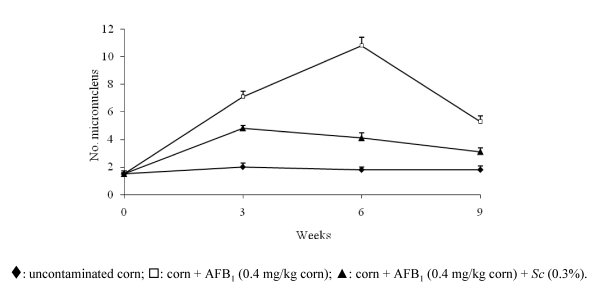
*Saccharomyces cerevisiae* (*Sc*) inhibitory effect on micronucleus frequency induced by aflatoxin B_1_ (AFB_1) _[58].

The experiment was similar to the ammonium one, except that its total duration was of nine weeks (six of treatment with the mutagen and antimutagen and three weeks of recovery with a diet free of these compounds). In this case, the antimutagen agent consisted of a 0.3% suspension of viable organisms of *Saccharomyces cerevisiae*. The frequency of MN was only observed at weeks 3, 6, and 9. The results showed a significant reduction since the third week on (50% approximately) and in the sixth week the inhibition reached 70%. A spontaneous recovery was observed in animals treated with AFB_1_ This recovery was of 60% in the last period. Furthermore, the use of probiotics reflected a recovery of 50% in the weight of the mice damaged by AFB_1_ (Figure 2). The antigenotoxic effect observed in our experiment is attributed to the *Sc* adsorbent capacity, particularly due to a chemical interaction between the mycotoxin and the components of the cell wall of yeast [22].

### 3.5. Protective Effect Caused by Constituents of Yeast Cell Wall against the DNA Damage Induced by AFB_1_

The yeast cell wall consists mainly of homopolysaccharides (mannans and glucans) and a minor proportion of heteropolysaccharides (glucomannans, galactomannans and xilomannans), proteins, chitin and lipids. There is evidence of the antimutagenic capacity of these oligosaccharides, specifically mannan (mannose with links α-1,6 and ramifications α-1,2 and α-1,3) and glucans (glucoses with links α-1,6 and ramifications β-1,2 and β-1,3) against antineoplastic compounds such as cyclophosphamide and mitomycin C [59,60,61].

Taking into account these antecedents and the properties mentioned above, our current researches are directed to determine the antigenotoxic capacity of mannan, glucan and glucomann and to decide if the mechanism is related to the formation of a molecular complex that diminishes the adsorption of the mutagenic agent. 

**Figure 3:**
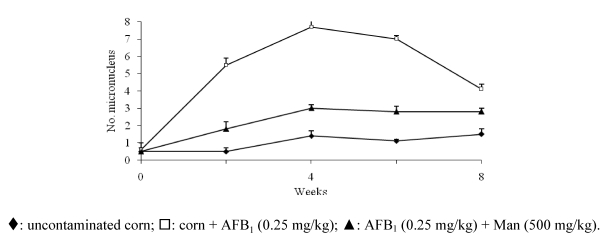
Mannan (Man) inhibitory effect on DNA damage on micronucleus frequency induced by aflatoxin B_1_ (AFB_1) _[58]_._

Recently, we completed a study of eight weeks with mannan following the model mentioned above: in the first period (four weeks), the animals consumed a balanced diet. They were fed with maize: (a) without AFB_1_; (b) with mannan (500 mg/kg); (c) with AFB_1_ (0.25 mg/kg), and (d) mannan (50, 250 and 500 mg/kg) plus AFB_1_ (0.25 mg/kg). In the last four weeks the mice consumed a diet free of antimutagen and mycotoxin. The evaluation of MN was made every week and showed a significant inhibition after the third week in mice treated with the highest dose of mannan, reaching the maximum antigenotoxic effect (50%) in the fourth week (Figure 3).

**Figure 4:**
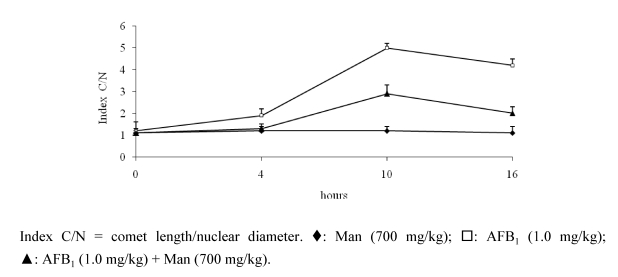
Mannan inhibitory effect on DNA damages induced by AFB_1 _in mice hepatocytes [58].

During the last four weeks, the animals showed a spontaneous recovery of approximately 70% [56]. To demonstrate directly the protection of mannan to DNA, we made a study using the single cell electrophoresis technique (comet assay) in hepatocytes of mice. Comet assay is a technique that can be applied practically to any cell suspension to determine breaks in the strings of DNA in individuals cells [62]. In our case, the procedure detected single-strand breaks. This experiment, unlike previous ones, was an acute one. The compounds were administered orally and the quantification of DNA damage was performed at 4, 10 and 16 hours. We included a control group treated with 700 mg/kg of mannan, a control group treated with 1.0 mg/kg of AFB_1_, and three groups in which we combined the mycotoxin (1.0 mg/kg) and mannan (100, 400 and 700 mg/kg). The results showed that the highest dose of mannan inhibited the genotoxicity caused by the mycotoxin since the first time, and the highest antigenotoxic effect was between 10 and 16 hours (approximately 60%) (Figure 4) [25]. These results corroborate the data previously obtained by SCEs and MN, and support the hypothesis of a possible chemical interaction between the compounds, which would cause a molecular complex that could be eliminated through the intestinal via without damaging the DNA. A partial damage was observed and it may be related to an amount of AFB_1_ absorbed and few adducts formed. Our hypothesis, suggests that this interaction would occur between the oligosaccharide hydroxide and the carbonyl group of AFB_1_, so, we actually analyzed this possibility by using the technique of attenuated total reflectance (ATR) in the intestine of animals treated with the same compounds. The incidence of a light beam of infrared frequency would show the functional groups of the probable chemical complex, which will be identified in the corresponding spectrum.

## 4. Generalities about the Ochratoxins

**Figure 5:**
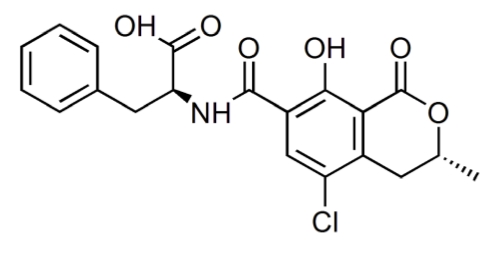
Chemical structure of ochratoxin A (OTA).

Ochratoxins are a group of structurally related metabolites that are produced by *Aspergillus ochraceus* and related species, as well as *Penicillium viridicatum* and other *Penicillium* species [63,64,65]. The main mycotoxin in this group is ochratoxin A (OTA) which also appears to be the only one of major carcinogenic significance. Ochratoxin B (OB), the decholoro derivate of OTA, is essentially non-toxic. Ochratoxin A was discovered as a metabolite of *Aspergillus ochraceus* in 1965 during a large screen of fungal metabolites that was designed specifically to identify new mycotoxins. Shortly thereafter, it was isolated from a commercial corn sample in the United States and recognized as a potent nephrotoxin [2]. Chemically, OTA comprises a polyketide-derived dihydroisocoumarin moiety linked via its 12-carboxyl group by an amide bond to L-β-phenylalanine (Figure 5). As with other mycotoxins, the substrate on which the molds grow as well as the moisture level, temperature (cold weather with low temperatures of 5 °C), and presence of competitive microflora interact to influence the level of toxin produced. The presence of OTA in several plants and animal products has been extensively reported [66,67,68,69,70]. OTA contamination is typically associated with grains stored in the mild weather of Europe and North America. This mycotoxin has been found in barley, oats, rye, wheat, coffee beans, and other plant products, with barley having a particularly high likelihood of contamination. There is also concern that ochratoxin may be present in certain wines, especially those from grapes contaminated with *Aspergillus carbonarius* [2]. The kidneys are the most susceptible organs to be contaminated by OTA. It can cause both acute and chronic kidney lesions. It principally operates in the first part of the proximal tubules in the kidney and induces a defect in the anion transport mechanism on the brush border of the proximal convoluted tubular cells and basolateral membranes [12,33].

## 5. DNA Damage Caused by OTA

Pfohl-Leszkowicz and Manderville [33] reviewed the toxicology of OTA and observed that following oxidative metabolism, forms a DNA-reactive quinone that can form guanine-specific DNA adducts. These adducts provide an important source of mutation. Very potent mutagenic effects can be shown in *S. typhimurium* TA 1535, 1538, and 98, providing certain preincubation protocols and types of metabolic activation are used. Ochratoxin A is genotoxic in *Escherichia coli* by means of induction of the SOS DNA repair activity. It is also mutagenic in NIH 3T3 cells expressing selected P450 cytochrome and carrying a shuttle vector containing the bacterial lacZ gene as a reporter gene [28]. An increase in sister chromatid exchange rates has been observed in CHO cells in presence of S9 mixture and in peripheral human lymphocytes cultured in a conditioned medium. Therefore, it induced DNA single-strand breaks in cultured mouse and CHO cells. Mammalian cells has been treated with a dose range of this toxin that would accumulate in single-strand DNA breaks, as revealed by the alkaline single-cell gel electrophoresis. This effect is greatly enhanced by the addition of an external metabolizing system in the form of a S9 mix from rat liver [33]. OTA induced a weak positive response for induction of unscheduled DNA synthesis in primary hepatocytes from ACI C3H strain mice and ACI strain rats. Dietary OTA induced renal adenomas and human hepatocellular carcinomas on mice and rats. Carcinogenic effects on humans are suspected because of the high incidence of kidney, pelvis, ureter, and urinary bladder carcinomas among patients suffering from Balkan endemic nephropathy. In regions with Balkan endemic nephropathy, high levels of OTA were found in human blood [2].

## 6. Ochratoxin A Decontamination Methods

Different studies in animal models have demonstrated that ochratoxin A is nephrotoxic, hepatotoxic, neurotoxic, immunotoxic, teratogenic, mutagenic and genotoxic [64,65,68,71,72]. Since 2002, the OTA has been classified by the International Agency for Research on Cancer as a carcinogenic agent for humans included in the group 2B [28,65,68]. Different strategies have been implemented to control and decontaminate food. In general, these methods have been classified as physical, chemical and biological (Table 3). In the case of the physical methods, milling is one of the most used because it reduces approximately 66% of the levels of OTA during the production of white wheat fluor [1]. Also, washing and brushing have shown good results in reducing the concentration of OTA below the permissible limits (Table 4) in sausage for human consumption [73]. It is important to remember that the permissible levels of OTA are different from country to country.

**Table 3:** Decontamination methods for ochratoxin A.

Methods	Technique	Example	Reference
Physical	Mechanical removal	Milling	[1]
		Washing and brushing	[73]
Chemical	Pesticides	Dinocap, Penconazole	[1]
	Fungicides	Iproidine, Azoxystrobin	[1]
	Essential oils	Oregano, Mint, Basil, Sage	[1]
	Gas treatment	Ozone	[74]
	Antifungals	Fusapyrone	[1]
Biological	Spores	Conidia	[75]
	Biodegradation	*Acinetobacter, Lactobacillus, Streptococcus thermophilus*, and *Bifidobacterium*	[1,76,77]

On the other hand, the chemical methods have been directed to the use of pesticides (like iproidine and azoxystrobin), because these substances can reduce by up to 80% the development of ochratoxin A produced by *A. ochraceus*. Essential oils of spices such as oregano, mint, basil, and sage have been effective used against damage produced by OTA. For example, oregano and mint can inhibit the development of this mycotoxin after 21 days of treatment, at concentrations of 100 ppm. There is evidence that treatment with ozone (O_3_) slightly decreases levels of OTA [1,74]. Several reports of OTA biodegradation have been published. *Streptococcus, thermophilus Bifidobacterium* and yogurt bacteria have completely reduced ochratoxin A levels in milk samples containing 0.05 and 0.1mg/L. Some strains of *A. fumigates*, *A. japonicus*, and *A. niger* have also been reported to be able to degrade OTA in products such as ochratoxin α [1,75,76,77,78].

**Table 4:** Permissible limits of ochratoxin A in several foods recommended in the European Union.

Food	Permissible limit (μg/kg)
Coffee beans	1.0–5.0
Instant coffee	0.8–10
Cereals	4.0–5.0
Table wine	2.0
Grape juice	2.0
Food for infants and childrens	0.5
Sausages	3.0
Dried fruits	0.2
Beer	3.0

### 6.1. Antimutagenic Strategies for the Control of Ochratoxin A Damage

Like decontamination methods for aflatoxins, the strategies to control the toxic effects of ochratoxin A have not been optimal, because they modify the nutritional values of food. Once again, the use of antimutagenic agents is another alternative to decrease this toxicity. The study is focused on analyzing the capacity of some substances to reduce the oxidative stress induced by the ochratoxin A.

Many reports have suggested the potential role for oxidative stress in OTA toxicity and carcinogenesis. In cell culture, OTA increases the DNA oxidation (generation of 8-OH-guanine) was correlated with a production of reactive oxygen species (ROS). Considering the role of ROS in chemically induced carcinogenesis, the ability of OTA to induce oxidative damage in cells may play an important role in OTA-induced carcinogenicity. Chemopreventive strategies designed to limit the several toxic effects caused by this mycotoxin are important public health goals in reducing the incidence of OTA induced neoplastic diseases [79].

### 6.2. *In Vitro* Studies

*In vitro* studies related to antimutagenesis began in the nineties. Initially, the experiments evaluated the possibility of success in microbial cultures and mammalian cell lines (Table 5). One of the first studies was conducted by Baudrimont *et al.* who analized the prevention of lipid peroxidation induced by OTA in Vero cells in culture by aspartame (L-aspartyl-L-phenylalanine methyl ester) a structural analogue of OTA and phenylalanine, piroxicam, a non steroidal anti-inflammatory drug and superoxide dismutase (SOD) + catalase (endogenous oxygen radical scavengers). The study found that in the presence of SOD + catalase, the malonaldehyde (MDA) production induced by OTA was significantly decreased. SOD and catalase, when applied prior to the mycotoxin, seemed to prevent lipid peroxidation more efficiently than piroxicam and aspartame. These molecules also partially prevented the OTA-induced leakage of MDA in the culture medium [80]. Another study was conducted by Turbic *et al*. These authors suggested that specific strains of lactic acid bacteria possessing antimutagenic properties can be used to remove the mutagenic contaminants of food, such as OTA [81]. More recently, studies with polyphenolic compounds (such as chrysin, quercetin, genistein, biochanin A), catechins, and rosmarinic acid have shown the same capacity [79,82,83,84].

### 6.3. *In Vivo* Studies

There is a variety of studies about the antimutagenic capacity of several substances. However, there are few experiments made with animals which demonstrate the potential protection of substances used against the damage produced by OTA. One of the earliest studies was made by Obrecht-Pflumio *et al.* (1996). These authors evaluated the protection capability of indomethacin and aspirin tested against the genotoxicity of ochratoxin A, particularly in the urinary bladder and kidney. The study showed that after a single oral administration of OTA to mice (2 mg/kg body weight) there existed a high level of DNA adducts detected in the urinary bladder. The study also demonstrated that two inhibitors of the prostaglandin H synthase, indomethacin and aspirin, administered before OTA treatment, dramatically reduced the amounts of DNA adducts, particularly in the urinary bladder and kidney. This suggests that protaglandin H synthase in the metabolism of OTA activates metabolites which react with DNA [85]. Recent studies (Table 6) are focused on analyzing substances in dietary honey, aspartame, Inula crithmoides extract, indomethacin, aspirin, and melatonin, and they generally measure glutathione (GSH), glutathione reductase (GR), glutathione peroxidase (GSPx), superoxide dismutase (SOD), catalase (CAT), glutathione-*S*-transferase (GST), lipid peroxidation (LPO), DNA adducts, and frequency of micronucleus [85,86,87,88,89,90].

**Table 5:** Principal antimutagenic studies *in vitro*.

Antimutagen	Cell line or strains of study	Inhibition%	Reference
Aspartame, phenylalanine, and piroxicam	Vero cells	50	[79]
*Lactobacillus rhamnosus*		76	[81]
Rosmarinic acid	Human hepatoma cells (Hep G2)	40	[84]
Chrysin, quercetin genistein, and biochanin A	Caco-2-cells	70	[83]
Epigallocathechin gallate, and epicatechin gallate	Pig kidney cells (LLC-PK1)	80	[79]
Gallic acid, vanillic acid, protocatechuic acid, caffeic acid, chlorogenic acid, and 4-hydroxybenzoic acid	Ochratoxigenic Aspergilli strains	50–60	[82]

**Table 6:** Summary of recent *in vivo* studies.

Year	Biological model	Evaluation parameters	Chemopreventive substance	Conclusion	Reference
1996	Mice	Quantification of aducts	Indomethacin and aspirin	These substances reduce the amounts of DNA adducts, particularly in the urinary bladder and kidney. This suggests a role of protaglandin H synthase in the metabolism of OTA leading to active metabolites which react with DNA	[85]
1998	Rats	Quantification of aducts	Aspartame	The molecular mechanism mediating the preventive effect of Aspartame is the delivery of phenylalanine by cleavage of the peptide and also the direct effect of the peptide on the bending capacity and transport of the toxin	[90]
2001	Rats	LPO, GSH, GR, GSPx, SOD, CAT, and GST	Melatonin (Mel)	Mel has a protective effect against OTA toxicity through an inhibition of the oxidative damage and stimulation of GST activities	[88]
2004	Rats	LPO, GSPx, CAT, and SOD	Melatonin (Mel)	Mel decreased the OTA-induced damage to support the antioxidant defense system and/or with free radical scavenger action	[87]
2006	Mice	Colonic probiotic bacteria, colon enzyme glucuronidases, and chromosomal aberrations	Dietary honey	Substituting sugars with honey in processed food can inhibit the harmful and genotoxic effects of mycotoxins, and improve the gut microflora	[86]
2008	Rats	Micronucleus	Inula crithmoides extract	The extract alone was successful in counteracting the oxidative stress and protect against the cytotoxicity produced by OTA	[89]

## 7. Conclusions

Finally, the information contained in this review suggests the probable application of different substances as an alternative method to avoid the toxicity produced by the AFB_1_ and the OTA in animals that have been fed with food contaminated with these mycotoxins. If the mycotoxicoses are reduced in animals dedicated to human consumption, the possibility of developing any neoplasy would diminish. This possibility is sustained by the information obtained from the substances that were analyzed as chemo-preventive and/or antigenotoxic and has contributed to develop the idea that cancer is susceptible of being detained in the predisplasic and displasic stages, that is to say, when the genetic changes are still reversible. Therefore, it is important and convenient to expand the surveys in order to confirm the antigenotoxic power of the substances analyzed in this review. The evaluation of other genotoxic parameters such as the sister chromatid exchanges, gene mutations, and single cell electrophoresis would be among them. Besides, it is necessary to establish clearly the anti-genotoxic action mechanisms of these and other substances.
